# The Effectiveness of Prophylactic Modified Neck Dissection for Reducing the Development of Lymph Node Recurrence of Papillary Thyroid Carcinoma

**DOI:** 10.1007/s00268-017-4023-6

**Published:** 2017-04-20

**Authors:** Yasuhiro Ito, Akira Miyauchi, Takumi Kudo, Minoru Kihara, Mitsuhiro Fukushima, Akihiro Miya

**Affiliations:** 1 Department of Surgery, Kuma Hospital, 8-2-35, Shimoyamate-dori, Chuo-ku, Kobe, 650-0011 Japan; 2 Department of Internal Medicine, Kuma Hospital, Kobe, Japan

## Abstract

**Aim:**

The most frequent recurrence site of papillary thyroid carcinoma (PTC) is the cervical lymph nodes. The introduction of an electric linear probe for use with ultrasonography in 1996 improved preoperative lateral neck evaluations. Before 2006, however, our hospital routinely performed prophylactic modified neck dissection (p-MND) for N0 or N1a PTCs >1 cm to prevent node recurrence. In 2006, we changed our policy and the indications for p-MND to PTCs >3 cm and/or with significant extrathyroid extension. Here, we retrospectively compared lymph node recurrence-free survival between PTCs with/without p-MND.

**Methods:**

We examined the cases of N0 or N1 and M0 PTC patients who underwent initial surgery in 1992–2012. To compare lymph node recurrence-free survival between patients who did/did not undergo p-MND, we divided these patients into three groups (excluding those whose surgery was in 2006): the 2045 patients whose surgery was performed in 1992–1996 (Group 1), the 2989 with surgery between 1997 (post-introduction of ultrasound electric linear probes) and 2005 (Group 2), and the 5332 operated on in 2007–2012 (Group 3).

**Results:**

The p-MND performance rate of Group 3 (9%) was much lower than that of Group 1 (80%), but the lymph node recurrence-free survival of the former was significantly better, probably due to differences in clinical features and neck evaluations by ultrasound between the two groups. Our analysis of the patients aged <75 years with 1.1–4-cm PTCs in Groups 2 and 3 showed that p-MND did not improve lymph node recurrence-free survival. p-MND did significantly improve lymph node recurrence-free survival for the extrathyroid extension-positive 3.1–4-cm PTCs, but not for the other subsets.

**Conclusions:**

Abolishing routine p-MND for PTCs in 2006 did not decrease lymph node recurrence-free survival, probably due to improved ultrasound preoperative neck evaluations and clinical feature changes. Selective p-MND for high-risk cases improved lymph node recurrence-free survival.

## Introduction

Papillary thyroid carcinoma (PTC) is the most common malignancy arising from thyroid follicular cells. Although PTCs are generally indolent lesions, cases showing certain characteristics are likely to recur and occasionally show a dire prognosis. PTCs frequently metastasize to the regional lymph nodes; in addition, the organ to which PTCs most frequently recurs is the cervical lymph nodes. The compartments of regional lymph nodes consist of central and lateral compartments. At the majority of Japanese healthcare facilities, the central compartment is routinely dissected even when no central node metastasis is detected on imaging studies, because it is not easy to avoid recurrent laryngeal nerve injury and permanent hypoparathyroidism in a reoperation for recurrence to this compartment. Regarding the lateral compartment for PTC cases, the Japanese guidelines do not clearly say whether or not a prophylactic modified neck dissection (p-MND) should be performed [1].

In the past, p-MND was frequently performed at many Japanese institutions in order to reduce the risk of PTC recurrence at the cervical lymph nodes, which are the most frequent site of recurrence for this lesion. This was the case at Kuma Hospital in Japan, where p-MND was routinely performed for PTCs >1 cm.

In regard to diagnosing lymph node recurrence, palpation had been the only method available until the late 1980s, when ultrasound was introduced as a useful tool for this purpose. Then, in 1996, use of an electric linear probe was added to further improve the diagnostic accuracy of the ultrasound. Fine-needle aspiration biopsy (FNAB) and thyroglobulin measurement of the washout of the needles used for FNAB have also contributed to these diagnoses [2].

In 2006, Kuma Hospital changed its policy, fundamentally limiting the indications for p-MND to PTCs >3 cm and/or with significant extrathyroid extension (Ex) (corresponding to T4a in the UICC/AJCC TNM classification [3]), based on our findings that tumor size >3 cm and Ex are the two major risk factors for lymph node recurrence [4]. Sugitani et al. [5] also demonstrated that p-MND can be considered for patients with tumors ≥4 cm or with distant metastasis at surgery. However, these studies were single-arm, and no comparative studies between patients who did and did not undergo p-MND have been published to date.

In the present study, therefore, we compared lymph node recurrence-free survival (LN-RFS) between PTC patients who did and those who did not undergo p-MND, using our hospital’s series of patients who underwent surgery during the eras of routine p-MND and selective p-MND, to determine whether p-MND is effective to reduce the development of lymph node recurrence in PTC patients.

## Patients and methods

### Patients

In 1996, we introduced an electric linear probe for use with ultrasonography at our hospital; this facilitated more accurate evaluations of both primary lesions and lymph node metastases. In 2006, we changed the indications for prophylactic MND for N0 or N1a patients as described above in the “Introduction” section. In the present retrospective analysis, we enrolled patients who underwent initial surgery for N0 or N1aM0 PTC between 1992 and 2014. All patients were diagnosed as negative for lateral node metastasis on preoperative imaging studies, predominantly on ultrasound. Our criteria for metastasis by ultrasound were as follows: (1) a clear hypoechoic pattern or dyshomogeneous pattern, with alternating hypoechoic and hyperechoic areas; (2) irregular cystic appearance; (3) presence of internal calcification; and (4) rounded or bulging shapes with increased anteroposterior diameter. Although these were similar to the criteria of Antonelli et al. [6], we did not adopt their criterion of suspicious nodes of size larger than 1 cm, which they included, because reactive nodes often exceed 1 cm in size. Instead, for suspicious nodes, we performed a fine-needle aspiration biopsy (FNAB) and measured thyroglobulin levels in the needle washout [2]. We excluded the patients who underwent their initial surgery in 2006 because we changed our p-MND policy during that year. We classified the enrolled patients into three groups based on the era of surgery: Group 1 consisted of the 2045 patients who underwent their initial surgery between 1992 and 1996; Group 2 of the 2989 patients who underwent the initial surgery between 1997 and 2005; and Group 3 of the 5332 patients who underwent the initial surgery between 2007 and 2014.

### Surgical designs

All of the patients underwent a thyroidectomy (lobectomy with isthmectomy, subtotal or total thyroidectomy) and therapeutic or prophylactic central node dissection. The indications for p-MND in the patients in the three groups are described above. All patients who underwent p-MND were dissected at levels IIA, III, and IV. Levels IIB and V were also dissected (partially or thoroughly) according to the discretion of the surgeon for a portion of patients. The extent of p-MND was not fundamentally changed throughout the three treatment eras in this study.

### Postoperative follow-up

Only 21 patients underwent radioactive iodine (RAI) ablation using 30 mCi RAI or more. Patients were followed up by ultrasound at least once per year to check whether lymph node recurrence appeared. FNABs and thyroglobulin measurements in the needle washout after FNAB for suspicious nodes were used to diagnose lymph node recurrence [2]. The median follow-up times in Groups 1, 2, and 3 were 222 months (9–353), 138 months (1–233), and 58 months (1–113), respectively.

### Statistical analysis

Variables were compared by the Mann–Whitney *U* test. The Kaplan–Meier method and log-rank test were used for the analysis of time-dependent variables. A *p* value <0.05 was regarded as significant. We used StatFlex software for these analyses.

## Results

Table 1 summarizes the clinicopathological features of the three patient groups. Over time, the incidences of small carcinomas (≤2 cm), older patients (≥55 years), male patients, and N1a cases increased and the incidence of Ex positivity decreased. Our series included no cases with extranodal tumor extension. Although the tumor sizes decreased over time, the incidence of clinical central node metastasis increased. However, the incidence of pathological central node metastasis decreased. We think that these phenomena were due to the decrease in tumor size and the increased sensitivity for the detection of node metastasis by the improved ultrasound examinations.

**Table 1:** Clinicopathological features of the PTC patients treated at Kuma Hospital, Japan in three treatment eras from 1992 to 2014

	Group 1^a^	Group 2	Group 3	*p* value
Age				
≥55 years	711 (35%)	1288 (43%)	2556 (48%)	<0.0001
<55 years	1334 (65%)	1701 (57%)	2776 (52%)	
Gender				
Male	128 (6%)	340 (11%)	821 (15%)	<0.0001
Female	1917 (94%)	2649 (89%)	4511 (85%)	
N1a				
Yes	44 (2%)	104 (4%)	369 (7%)	<0.0001
No	2045 (98%)	2885 (96%)	4963 (93%)	
Pathological central node metastasis				
Yes	1279 (66%)	1572 (54%)	2499 (47%)	<0.0001
No	660 (34%)	1324 (46%)	2833 (53%)	
Unknown	106	93	0	
Tumor size				
>4 cm	202 (10%)	164 (5%)	18 (0.3%)	<0.0001
3.1–4 cm	266 (13%)	242 (8%)	30 (0.6%)	
2.1–3 cm	491 (24%)	546 (18%)	752 (14%)	
≤2 cm	1086 (53%)	2037 (69%)	4098 (77%)	
pN1b				
Yes	1010 (62%)	988 (54%)	321 (66%)	0.1284
No	622 (38%)	848 (46%)	164 (34%)	
Unknown	413	1153	4847	
Ex**				
Yes	182 (9%)	263 (9%)	374 (7%)	<0.0001
No	1853 (91%)	2726 (91%)	4958 (93%)	
Total	2045 (100%)	2989 (100%)	5332 (100%)	

** Significant extrathyroid extension

^a^Group 1: Patients who underwent initial surgery in 1992–1996; Group 2: Patients who underwent initial surgery in 1997–2005; Group 3: Patients who underwent initial surgery in 2007–2014

Ipsilateral or bilateral p-MND was performed for 1632 patients (80%) in Group 1, 1835 patients (61%) in Group 2, and only 485 patients (9%) in Group 3. The performance rate of p-MND in Groups 1 and 2 was significantly (*p* < 0.0001) higher than that in Group 3. To date, 110, 100, and 63 patients showed recurrence in the regional lymph nodes in Groups 1, 2, and 3, respectively. Recurrence in distant organs such as the lung, bone, and liver was detected in 33, 17, and 7 patients in Groups 1, 2, and 3, respectively. To determine the complete number of patients who died of PTC, we sent a questionnaire to the patients who were referred to other hospitals for postoperative follow-up. To date, 7, 9, and 8 patients have died of PTC in Groups 1, 2, and 3, respectively.

The average numbers of harvested lateral lymph nodes were 21.4 in Group 1, 13.9 in Group 2, and 14.1 in Group 3, respectively. The number of harvested nodes in Group 1 was significantly larger than the numbers in Groups 2 and 3 (*p* < 0.0001). The average numbers of metastatic nodes in the lateral compartment in Groups 1, 2, and 3 were 2.6, 1.6, and 2.0, respectively. The number of metastatic nodes in Group 1 was significantly larger than the numbers of metastatic nodes in Group 2 (<0.0001) and Group 3 (*p* = 0.0467).

**Fig. 1:**
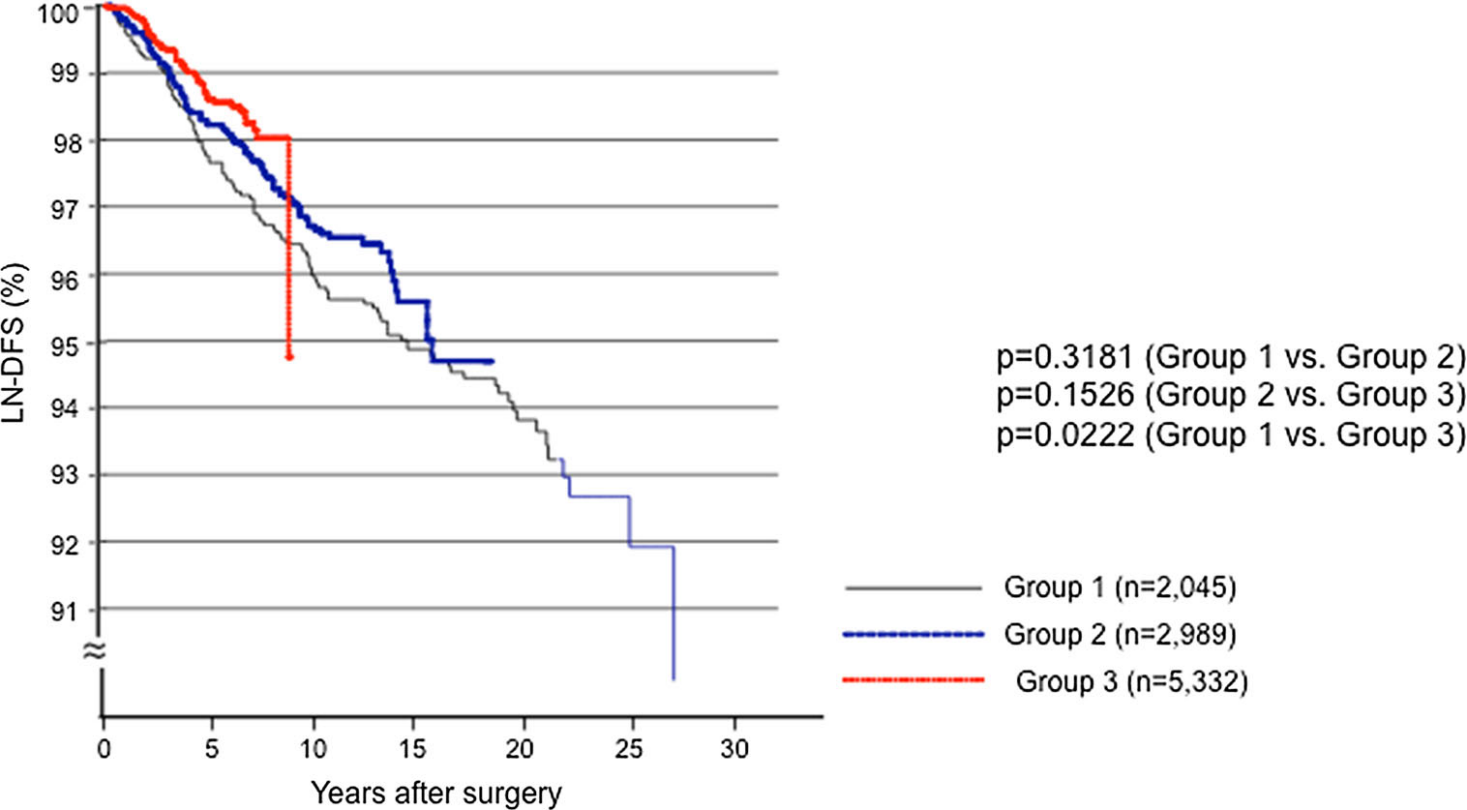
Kaplan–Meier curves for the lymph node recurrence-free survival (LN-RFS) of the patients in Groups 1, 2, and 3

We then compared the LN-RFS of the three patient groups. The LN-RFS between Groups 1 and 2 and between Groups 2 and 3 did not differ significantly, but the LN-RFS of Group 3 was significantly better (*p* = 0.0222) than that of Group 1 (Fig. 1).

**Fig. 2:**
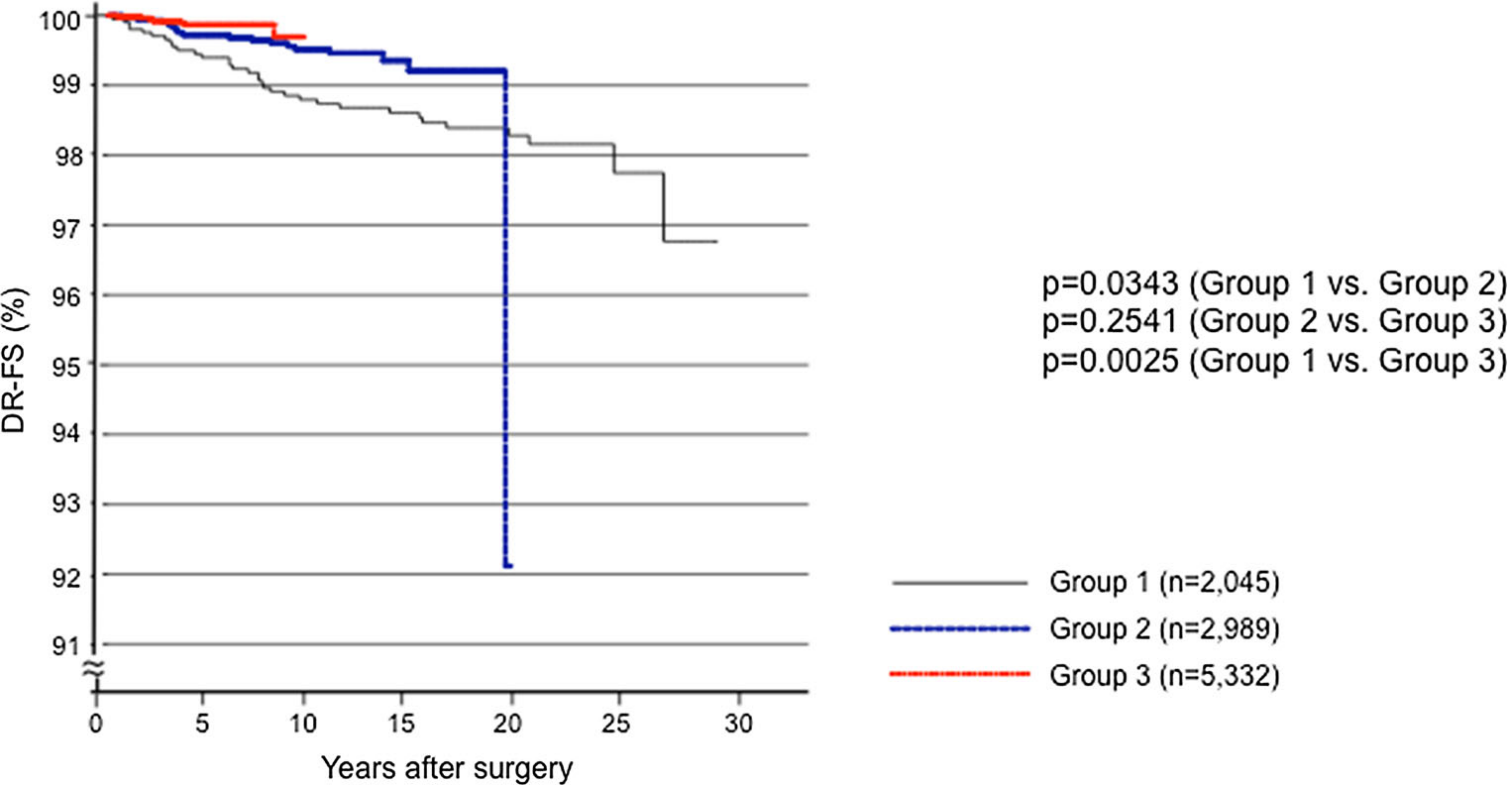
Kaplan–Meier curves for the distant recurrence-free survival (DR-FS) of the patients in Groups 1, 2, and 3

Figure 2 illustrates the distant recurrence-free survival (DR-FS) of the three groups. The DR-FS rates were significantly better in Groups 2 and 3 compared to Group 1 (*p* = 0.0343 and *p* = 0.0025, respectively).

We conducted a further analysis regarding lymph node recurrence in only the Group 2 and 3 patients, because of the introduction of the ultrasound electric linear probe in 1996, which enabled us to evaluate lymph node metastasis more accurately. We excluded patients with tumors ≤1 cm because most of these patients did not undergo p-MND. PTCs >4 cm in size were also excluded because most of these patients underwent p-MND. Finally, we excluded the patients ≥75 years old because most of them did not undergo p-MND due to their advanced age.

**Fig. 3:**
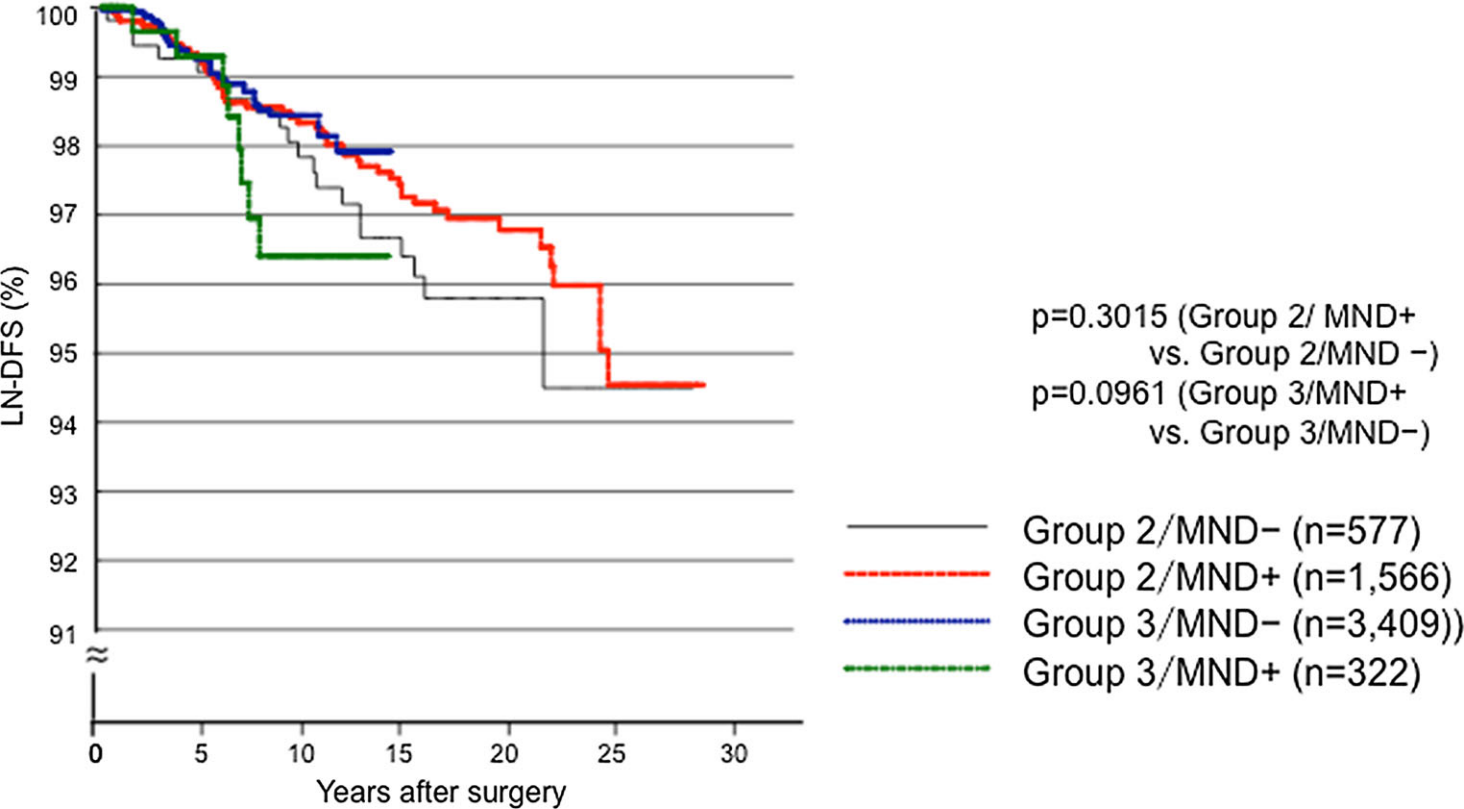
Kaplan–Meier curves for the LN-RFS of patients aged <75 years with 1.1–4 cm PTCs in Groups 2 and 3 who did or did not undergo a p-MND

Tables 2 and 3 summarize the backgrounds and clinicopathological features of the patients in Groups 2 and 3 examined in this analysis who did or did not undergo p-MND. The patients who underwent p-MND were younger, more frequently pathological central node-positive and Ex-positive, and had larger tumors. The difference in the incidence of large tumors and Ex positivity between the patients with and without p-MND was clearer in Group 3 than in Group 2, because we fundamentally limited the indications for p-MND in the Group 3 patients to tumors >3 cm or positive for Ex. Figure 3 shows the LN-DFS of the Group 2 and 3 patients who did and did not undergo p-MND. In both groups, p-MND did not improve the LN-DFS of patients.

**Table 2:** Clinicopathological features of the patients with PTCs 1.1–4 cm and aged <75 years in Group 2 who did or did not undergo p-MND

	p-MND (+)	p-MND (−)	*p* value
Age			
≥55 years	655 (42%)	286 (49%)	0.0015
<55 years	911 (58%)	292 (51%)	
Gender			
Male	177 (11%)	57 (10%)	0.3485
Female	1389 (89%)	520 (90%)	
N1a			
Yes	72 (4%)	20 (3%)	0.2571
No	1494 (96%)	557 (97%)	
Pathological central node metastasis			
Yes	949 (62%)	272 (49%)	<0.0001
No	591 (38%)	283 (51%)	
Unknown	26	22	
Tumor size			
3.1–4 cm	234 (15%)	21 (4%)	<0.0001
2.1–3 cm	492 (31%)	75 (13%)	
1.1–2 cm	840 (54%)	481 (83%)	
pN1b			
Yes	830 (53%)		
No	736 (47%)		
Unknown		577	
Ex			
Yes	187 (12%)	32 (6%)	<0.0001
No	1379 (88%)	545 (94%)	
Total	1566 (100%)	577 (100%)

**Table 3:** Clinicopathological features of the patients with PTCs 1.1–4 cm and aged <75 years in Group 3 who did or did not undergo p-MND

	p-MND (+)	p-MND (−)	*p* value
Age			
≥55 years	146 (45%)	1786 (52%)	0.0155
<55 years	176 (55%)	1623 (48%)	
Gender			
Male	60 (19%)	529 (16%)	0.1427
Female	262 (81%)	2880 (84%)	
N1a			
Yes	277 (86%)	3204 (94%)	<0.0001
No	45 (14%)	205 (6%)	
Pathological central node metastasis			
Yes	221 (69%)	1614 (47%)	<0.0001
No	101 (31%)	1795 (53%)	
Tumor size			
3.1–4 cm	151 (47%)	184 (5%)	<0.0001
2.1–3 cm	91 (28%)	726 (21%)	
1.1–2 cm	80 (25%)	2499 (73%)	
pN1b			
Yes	208 (65%)		
No	114 (35%)		
Unknown		3409	
Ex			
Yes	70 (22%)	246 (7%)	<0.0001
No	252 (78%)	3163 (93%)	
Total	322 (100%)	3409 (100%)

**Fig. 4:**
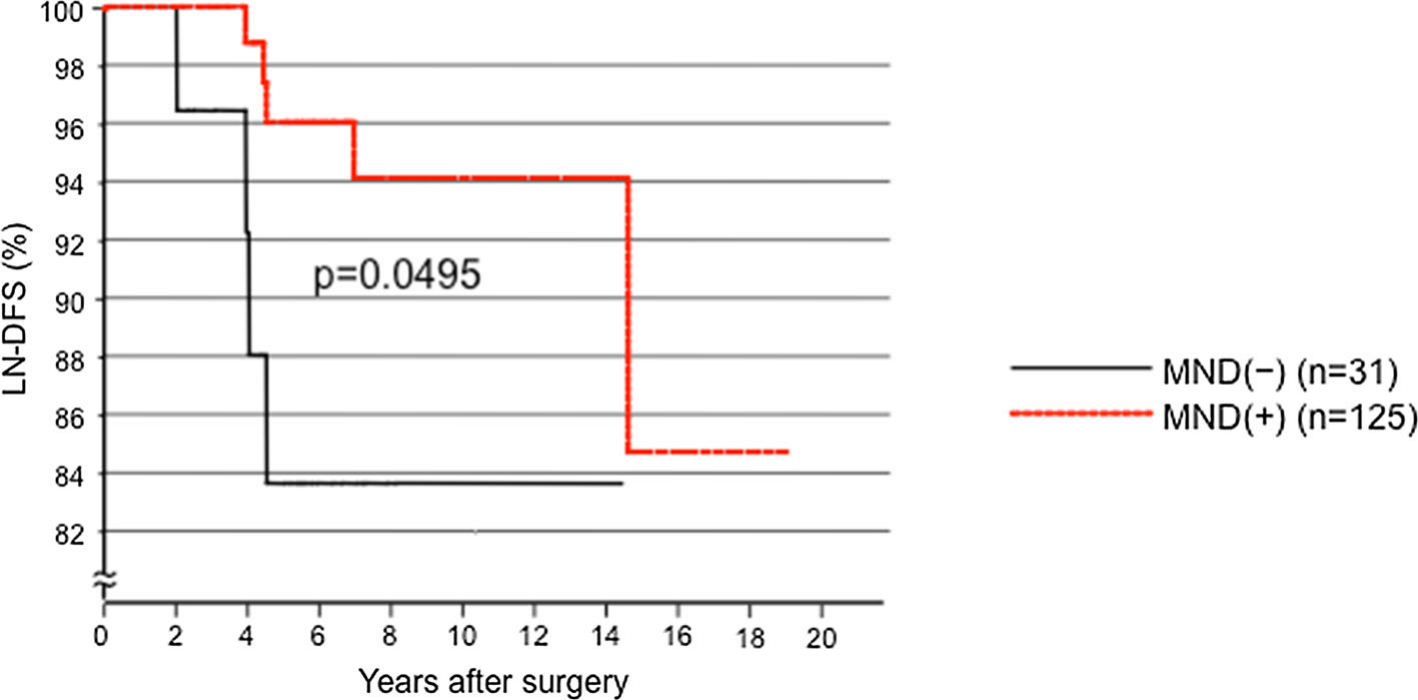
Kaplan–Meier curves for the LN-RFS of the patients aged <75 years with PTCs measuring 3.1–4 cm and positive for Ex in patients who underwent their initial surgery between 1997 and 2012

For subset analyses, we enrolled patients aged <75 years with tumors >1 and <4 cm who underwent an initial surgery between 1997 and 2012 (here we included the patients who underwent surgery in 2006: 404 patients, 112 of whom underwent p-MND). We performed the subset analyses by examining pairs of the following factors: patients’ age (cutoff: 55 years), tumor size (cutoff: 3 cm), Ex, and N1a. As shown in Fig. 4, in the subset of tumors in Groups 2 and 3 measuring 3.1–4 cm and with positive Ex, the LN-RFS was significantly better (*p* = 0.0495) in the patients who underwent p-MND than in patients who did not undergo p-MND. In other subsets, p-MND did not improve the LN-RFS of the patients (data not shown).

## Discussion

In Japan, p-MND was previously performed in an almost routine fashion to reduce the risk of PTC recurrence to the lymph nodes, because PTC very frequently recurs in the regional lymph nodes. Indeed, the incidence of lateral node metastasis is very high in PTC, and the specificity and negative predictive values of lateral node metastasis at a preoperative ultrasound examination in N0 or N1a patients are very low [4], indicating that lateral node metastases frequently remain undissected if p-MND is not performed.

Our present study is the first to compare the recurrence of PTC at the lymph nodes in a large series of patients who did or did not undergo p-MND. Our analyses demonstrated that (1) the LN-RFS of Group 1 did not differ from or was even worse than those of Groups 2 and 3, even though the percentage of patients undergoing p-MND was higher in Group 1, and (2) in PTCs ≤4 cm, p-MMD did not improve the LN-RFS of the patients except for those with tumors 3.1–4 cm and extrathyroid extension.

Although the percentage of patients who underwent p-MND in Group 1 was the highest of the three groups, the Group 1 patients were more likely to have lymph node recurrence than the Group 3 patients. Because the 1996 introduction of the electric linear probe for ultrasonography facilitated much more accurate evaluations of primary lesions and lymph node metastases, many Group 1 patients who would have been classified as N1b at presentation and diagnosed as lateral node-positive before 1996 were thus included in Group 1 in the present study. We compared the numbers of metastatic nodes and harvested nodes among these three groups, and found that both parameters were significantly higher in Group 1 than in Groups 2 and 3. Although the extent of p-MND was not fundamentally changed across the three treatment eras, p-MND might have been more actively performed in the Group 1 than in the Group 2 or Group 3 treatment era. Nonetheless, the rate of lymph node recurrence was higher in Group 1 patients than in Group 2 or 3 patients. This also supports the notion that a considerable number of cases regarded as high-risk (N1b) were diagnosed as N0 or N1a in Group 1. In addition, the incidences of PTCs >2 cm, N1a, and Ex positivity, which are risk factors for PTC recurrence [7, 8], were higher in Group 1 than the other groups. These findings may explain the likeliness of lymph node recurrence in the Group 1 patients. The likeliness of distant recurrence in our Group 1 patients should also be considered to be due to the involvement of aggressive cases in this group.

Next, we compared the LN-RFS between the Group 2 and 3 patients aged <75 years with tumors measuring 1.1–4 cm who did or did not undergo p-MND. At the time of surgery for the Group 2 patients, p-MND was frequently performed. Although whether or not p-MND is performed depends on the discretion of the surgeon, the Group 2 patients with large tumors and Ex positivity more frequently underwent p-MND (as shown in Table 2). Since large tumor size and Ex positivity are known as predictors of lymph node recurrence [4], it appears that our Group 2 patients at risk of lymph node recurrence more frequently underwent p-MND based on the discretion of their surgeons. However, in Group 2, the patients who underwent p-MND did not have better LN-RFS than the patients who did not. This may have been due to the selection of higher-risk patients for p-MND.

The percentage of patients in Group 3 who underwent p-MND was significantly decreased compared to Group 2, because we fundamentally narrowed the indications for p-MND to patients with large tumors (>3 cm) and/or Ex positivity. However, even under these conditions, the LN-RFS did not differ significantly between the patients who did and those who did not undergo p-MND. Our subset analyses showed that p-MND improved the LN-RFS of patients with PTCs measuring 3.1–4 cm and Ex positivity. However, for other subsets, p-MND did not help to prevent lymph node recurrence.

MND can induce various complications such as Horner syndrome, chyle leakage, and injury of the jugular vein and vagus nerve [9], although these are uncommon events in p-MND cases. In addition, thyroid surgery patients who undergo MND experience neck pain, discomfort, and stiffness more often and more strongly than those who do not undergo MND, although rehabilitation can reduce these symptoms to some extent [10]. We thus conclude that p-MND should not be routinely recommended for N0 and N1a patients with PTCs ≤4 cm, and we propose that the indications for p-MND should remain very limited.

Several limitations of the present study bear mention. This was not a prospective study, and the decision regarding whether or not a patient would undergo p-MND was not random. In addition, p-MND is still performed almost routinely for PTCs >4 cm at our hospital, and we could not perform a comparative study for the subset of patients with PTCs >4 cm. It thus remains unclear whether p-MND is beneficial for PTCs >4 cm.

In summary, p-MND for N0 or N1a PTCs measuring ≤4 cm did not improve the lymph node recurrence-free survival of the patients except for those with PTCs measuring 3.1–4 cm and with significant extrathyroid extension, indicating that the contribution of p-MND to the improvement of these patients’ prognoses is very limited. Further studies are needed to determine whether p-MND contributes to the improvement of the prognosis of patients with PTCs >4 cm.
